# Cumulative damage characteristics of tunnel initial support concrete under blasting load

**DOI:** 10.1038/s41598-024-84032-9

**Published:** 2025-01-23

**Authors:** Yan Zhao, Zhuang Zhang, Lijie Ge, Lingling Xu, Huadong Zhao, Mingzan Yu

**Affiliations:** 1 Hebei University of Architecture, Hebei, 075000 China; 2 School of Mechanics and Civil Engineering, China University of Mining and Technology (Beijing), Beijing, 100083 China

**Keywords:** Sonic testing, Blasting vibration monitoring, Blasting load, Cumulative damage, Initial support, Control threshold, Tropical ecology, Hydrology

## Abstract

Relying on the Beijing-Zhangjiakou high-speed railway Cao Mao Shan tunnel project, blasting vibration monitoring and sound wave testing experiments were carried out. The monitoring results show that the blasting vibration velocity corresponding to the initial support satisfies the Sadowski formula. The results of the sonic test show that with the increase of blasting times, the cumulative damage increases gradually, but the blasting damage increment shows a downward trend. In addition, as the blasting distance decreases, the blasting cumulative damage effect is significant. Through data analysis and curve fitting, the cumulative damage range *R*_*cr*_ and critical blasting vibration velocity *PPV*_*cr*_ corresponding to blasting construction are obtained respectively. The numerical analysis results show that there is a good exponential function relationship between the cumulative damage range *R*_*cr*_ and the corresponding critical blasting vibration velocity *PPV*_*cr*_. The purpose of quantitative control of blasting damage can be achieved by setting the corresponding vibration velocity threshold.

## Introduction

Blasting construction has the advantages of simple operation, good economic benefits and fast construction progress, so it is widely used in transportation infrastructure, municipal engineering and metal mining. For tunnel projects, especially mountain tunnel projects, blasting construction technology^1–3^ is still the main method for excavation and advancement. In general highway and railway tunnel projects, the composite support system consisting of anchor spraying support and integral cast-in-place concrete is the main form of lining structure. The chemical energy released instantly during blasting not only breaks the rock mass, but also diffuses to the surrounding area in the form of stress waves^4–8^. The tunnel lining structure, especially the initial support close to the blast source, is greatly affected by the blasting vibration. In addition, under the action of multiple blasting vibration loads, microscopic damage or even macroscopic cracking will inevitably occur inside the initial support concrete of the tunnel, weakening its structural bearing capacity^9–11^. Therefore, it is necessary to conduct a systematic study on the cumulative damage effect of the initial support of the tunnel under the action of cyclic blasting dynamic loads.

In the past 30 years, many scholars^12–14^ have introduced the concept of damage variables based on the knowledge of continuum damage mechanics and formed a series of theoretical models of blasting cumulative damage through mathematical statistics or numerical simulation. D.E.Crady et al.^15^ believed that the number of newly generated cracks under blasting obeys the exponential distribution law. Based on indoor model experiments, L.M.Taylor et al.^16^ established the functional relationship between damage variables and elastic modulus and crack distribution density. T. Kawmaoto et al.^17^ considered engineering rock mass as a continuous material with original defects and believed that there is a close relationship between the macroscopic defects and microscopic damage of the rock mass. In other words, the damage variable is an important intermediate link between the macroscopic defects and microscopic damage of the engineering rock mass. A.M.Rubin et al.^18^ found that the longitudinal wave velocity inside the rock mass is related to the degree of development of its internal cracks, and the degree of blasting damage to the rock mass can be evaluated by the change in the longitudinal wave velocity inside the rock mass. In addition, the K-G model, TCK model and Yang-Liu model are also widely used in the study of rock damage.

The above studies are centered on the damage of surrounding rocks under blasting dynamic loads, but there are few studies on the damage effects of tunnel lining structures, especially on the initial support. Based on a large indoor model experiment, Shan et al.^19^ discussed the destructive effect of tunnel blasting on freshly shotcrete by using the acoustic wave testing method. Based on the results of ultrasonic testing, Zhu et al.^20^ studied the cumulative damage effect of secondary lining concrete under multiple blasting and established a functional relationship between the cumulative blasting damage and the number of blasting cycles. From the perspective of blasting stress wave transmission, Chen et al.^21^ studied the contribution of blasting stress P wave and S wave to lining concrete damage. The above studies on the damage characteristics of lining concrete were all obtained through acoustic wave testing. However, due to the influence of the acquisition environment during on-site monitoring, acoustic wave testing often encounters certain operational difficulties. Compared with sonic wave testing, blasting vibration monitoring is simple and easy, and it is less affected by the environment. Therefore, many relevant specifications or studies^22–24^ used the critical particle peak velocity of blasting damage corresponding to engineering rock mass as the safety criterion for cumulative damage of rock mass blasting, and have achieved good application results. As shown in Table 1, Bauer and Calder^25^ divided the *PPV* thresholds of different rock mass blasting damage according to the degree of rock mass damage.

**Table 1:** *PPV* thresholds for rock blasting damage.

PPV/cm·s^-1^	Effects of rock mass damage
< 25	Intact rock will not fracture
25 ~ 63.5	The rock mass will undergo slight tensile cracking
63.5 ~ 254	Severe tensile cracks and radial cracks
> 254	The rock mass is completely broken

Mojitabai and Beattie^26^ defined the critical value of *PPV* for engineering rock mass based on the mineral composition of the rock, as shown in Table 2.

**Table 2:** *PPV* thresholds of rock blasting damage under different mineral composition conditions.

Types of rocks	σ_c_ /MPa	RQD/%	PPV/cm·s^-1^		
			Minor injury area	Moderate damage area	Serious injuries
Soft gneiss	14 ~ 30	20	13 ~ 15.5	15.5 ~ 35.5	>35.5
Hard gneiss	49	50	23 ~ 35	35 ~ 60	> 60
Shultze granite	30 ~ 55	40	31 ~ 47	47 ~ 170	> 170
Phenocryst granite	30 ~ 85	40	44 ~ 77.5	775 ~ 1240	> 1240

Based on the Damaoshan Tunnel project, Xia et al.^27^ systematically studied the damage effect of blasting in a newly built tunnel on the lining structure of the adjacent existing tunnel, and proposed a blasting damage control method based on *PPV*. Cao et al.^28^ used LS-DYNA to study the cumulative damage effect of cutting blasting on interlayers in parallel tunnels and established a mathematical relationship between the maximum segment charge, damage range and critical *PPV*. For blasting excavation projects on rock slopes, Yang et al.^29^ investigated the development of blasting damage variables along the slope surface through acoustic wave testing and numerical simulation. Due to the influence of explosion shock waves and flying rocks, it is not realistic to arrange blasting vibration monitoring instruments at the critical damage boundary. Therefore, Yang set the *PPV*-related threshold of the upper slope to achieve the purpose of controlling slope damage. In the Guangdong Aoling Nuclear Power Plant Phase II foundation blasting excavation project, Xia et al.^30^ studied the intrinsic relationship between the transmission of blasting vibration waves and foundation blasting damage variables by combining on-site acoustic wave testing with FLAC^3D^ simulation. Field engineering practice shows that the *PPV* of the rock mass 30 m away from the explosion source can be used as the only indicator to control the blasting damage range of the nuclear power plant foundation. Xia et al.^31^ conducted a special case study on blasting damage of columnar jointed basalt at the Baihetan dam site. The results showed that the *PPV* critical threshold can be considered as an inherent property of the engineering rock mass and is not affected by the blasting construction method or the detonation method. So, similar to the treatment method for rock mass, can the same treatment idea be used for the study of blasting damage in the initial support of tunnels? This is a question worth thinking about and studying.

Relying on the Caomaoshan Tunnel Project of Beijing-Zhangjiakou High-Speed ​​Railway, this paper mainly studies the blasting vibration effect and cumulative damage characteristics of the initial support concrete under tunnel blasting construction condition. By introducing the concept of cumulative damage variables, the functional relationship between the critical distance of blasting damage and the corresponding *PPV* threshold is studied, in order to achieve the purpose of quantitatively controlling the cumulative damage range of initial support blasting.

## Project background and monitoring plan

### Project background

**Fig. 1:**
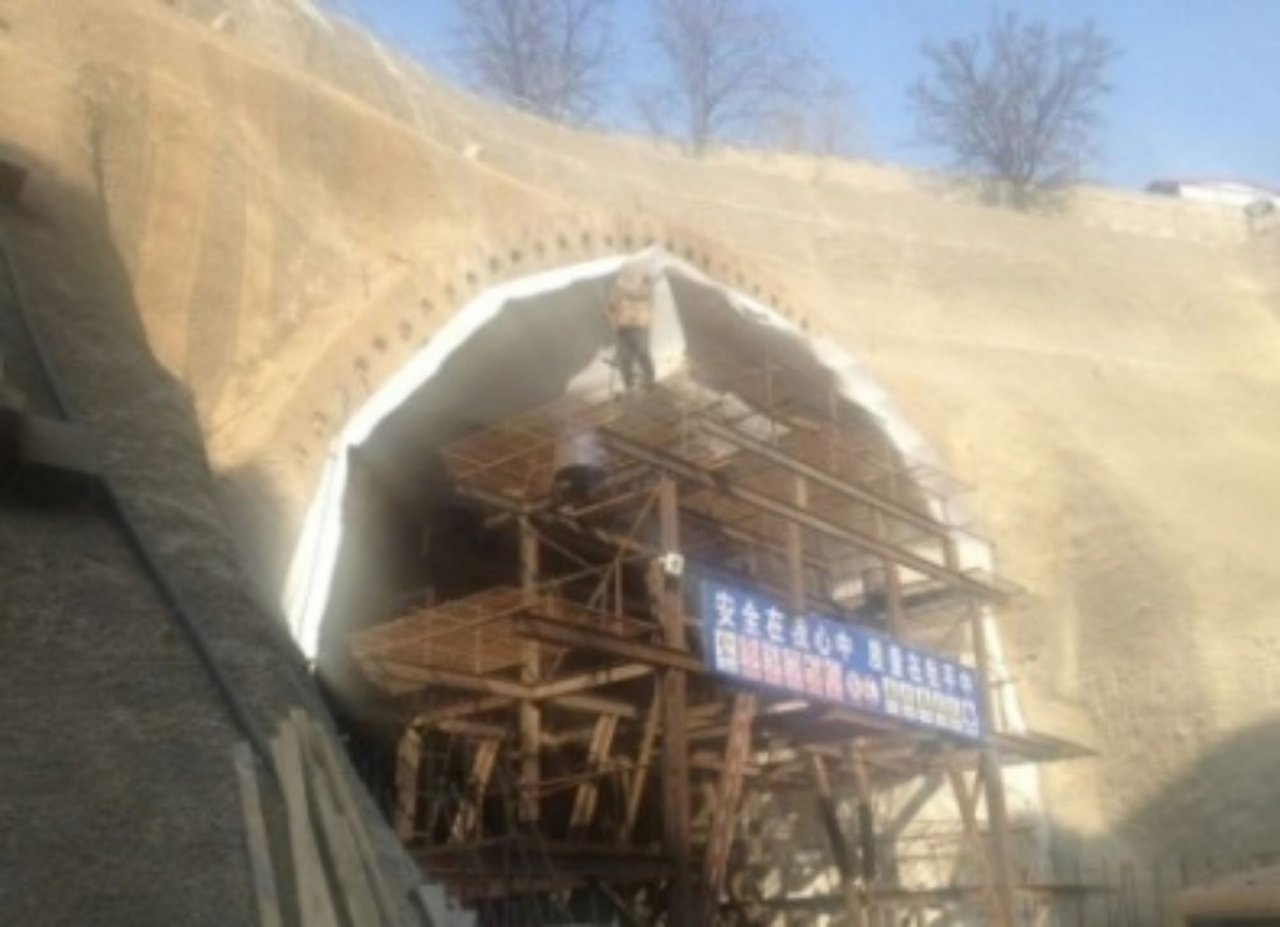
Site layout of the tunnel entrance area^33^.

The Beijing-Zhangjiakou high-speed railway starts from Beijing North Station, with stations such as Qinghe, Badaling Great Wall, Xiahuayuan, Xuanhua North, etc. along the way, and ends at Zhangjiakou Station. The railway is 174 km long and is one of the important channels connecting Beijing with Shanxi and Inner Mongolia. Caomaoshan Tunnel^32^ is a key control project of the Beijing-Zhangjiakou High-speed Railway, located in Chenjiazhuang Village, Xuanhua District, Zhangjiakou City, Hebei Province. The new tunnel adopts a single-hole double-track mode with a total length of 7,340 m. The tunnel passes through the main vein of Caomaoshan, with a maximum burial depth of about 109 m. The tunnel passes through the new loess and silty clay strata of the Upper Pleistocene of the Quaternary System. The fully weathered tuff it passes through has moderate expansion, which may cause accidents such as water inrush, mud inrush, surrounding rock collapse and large deformation, posing a major safety hazard to the blasting construction in this area of the tunnel. The tunnel entrance section is mainly composed of Grade III surrounding rock. Figure 1 is the layout of the Caomaoshan Tunnel entrance work area.

**Fig. 2:**
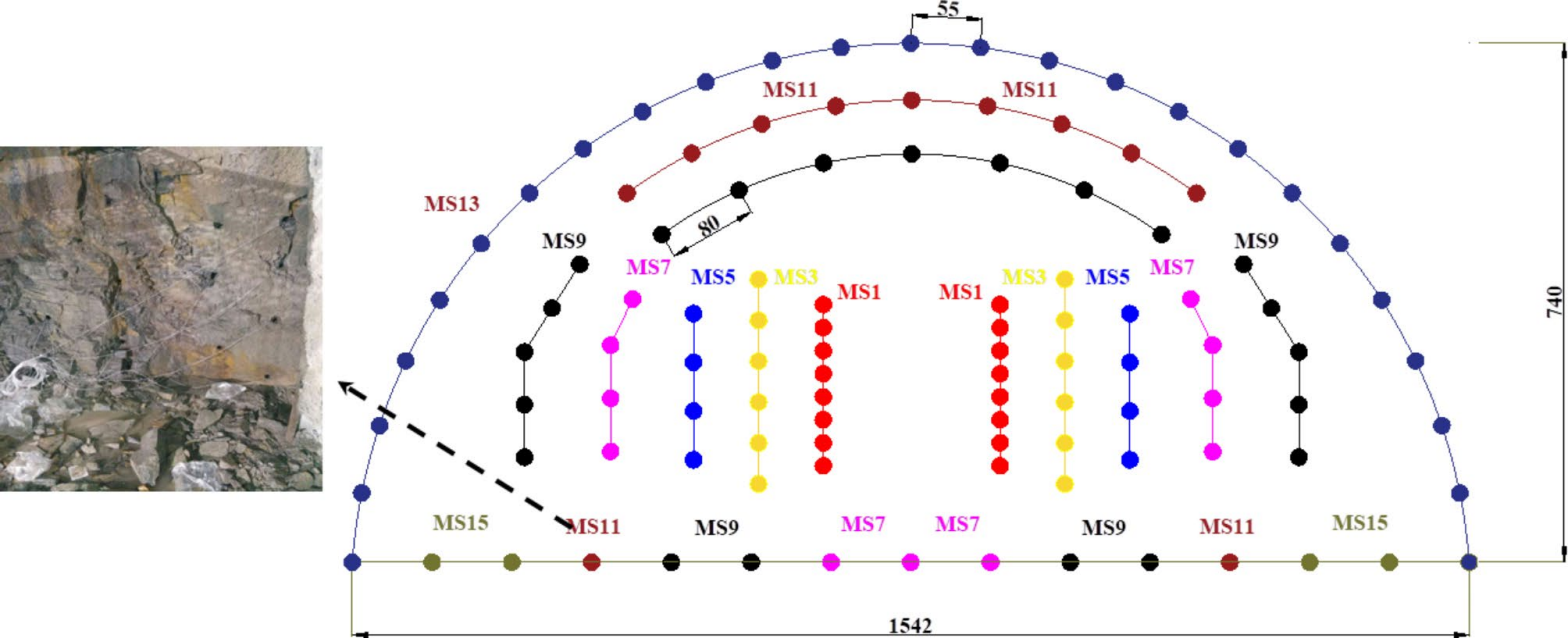
Layout of the upper steps of the tunnel. Note: The numbers in the figure are in centimeters.

According to the tunnel surrounding rock grade and geological conditions, the tunnel entrance section is excavated mainly using the step method, and the amount of explosives used in the blasting construction process is strictly controlled. The blasting excavation depth is controlled within the range of 2.0 m ~ 3.0 m. The blasting construction uses 2# rock emulsion explosive, the diameter of the blast hole is 42 mm, and the diameter of the charge roll is 32 mm. Tunnel blasting uses an axially uncoupled spacing method to install explosives and electronic detonators for detonation. The length of the gun mud filling is not less than 0.3 m. A large amount of explosives is used for blasting on the upper steps of the tunnel, which causes greater blasting vibration. The arrangement of blastholes on the upper steps of the tunnel is shown in Fig. 2, and the specific charge amount and blasting construction parameters are shown in Table 3.

**Table 3:** Specific charge for tunnel blasting.

Type of blasthole	Detonator level	Delay time/ms	Number of blast holes	Charge quantity for a single blast hole/ kg	Single stage charge/ kg
Cutting hole	MS1	25~45	16	2.7	43.2
Auxiliary hole	MS3	55~85	12	1.5	18
Auxiliary hole	MS5	75~105	8	2.1	16.8
Auxiliary hole	MS7	85~115	11	1.2	13.2
Auxiliary hole	MS9	90~125	21	1.2	25.2
Auxiliary hole	MS11	95~130	11	1.2	13.2
Peripheral hole	MS13	100~140	25	0.6	15
Floor hole	MS15	100~150	4	1.2	4.8
Subtotal			108		149.4

According to the on-site construction plan, the initial support of the tunnel is mainly an anchor-sprayed structure consisting of shotcrete combined with wire mesh, locking anchor rods, etc. During the on-site construction process, steel plates are used to reinforce the locking anchor rods and form a “U”-shaped connection with the steel frame. In addition, the thickness of the shotcrete is 12 cm. The length of the anchor inserted into the rock mass is 4.5 m and the diameter is 32 mm.

### On-site monitoring test plan

**Fig. 3:**
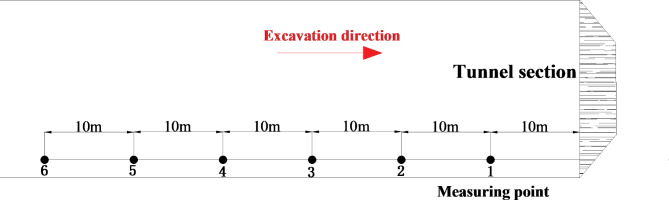
Plane layout of monitoring points.

The on-site test content mainly includes two main parts: blasting vibration monitoring and blasting sound wave testing. The blasting vibration monitoring uses the TC-4850 blasting vibration meter developed by Zhongke Measurement and Control Co., Ltd. The blasting vibration meter is equipped with three specific channels, which can simultaneously collect the blasting vibration speed and vibration frequency in three mutually perpendicular directions. The blasting sound wave test uses the RSM-SY5 intelligent sound wave detector developed by the Wuhan Institute of Rock and Soil Mechanics, Chinese Academy of Sciences. Considering the content of this test, the acoustic wave test was carried out using the flat measurement method, that is, the test transducer was arranged on the surface of the initial support of the tunnel. As shown in Fig. 3, a total of 6 measuring points were arranged in this field test. During the first blasting construction, measuring point 1 was 10 m away from the tunnel face, and the adjacent spacing of other measuring points was 10 m. As the tunnel section is excavated, the distance between the monitoring point and the explosion source changes accordingly. During the on-site monitoring process, the blasting vibration sensor is fixed to the initial support surface of the tunnel through the configured stainless steel clips, so that each sensor is close to the outer surface of the tunnel lining. In addition, each vibration sensor is 2.5 m away from the bottom of the tunnel. Before the experiment, a homemade steel cage was placed outside the vibration meter to prevent it from being damaged by rocks thrown during the blasting process. During the field test, the sensor’s X direction was toward the tunnel face, the Y direction was toward the inside of the tunnel diameter, and the Z direction was perpendicular to the XY plane and upward.

**Fig. 4:**
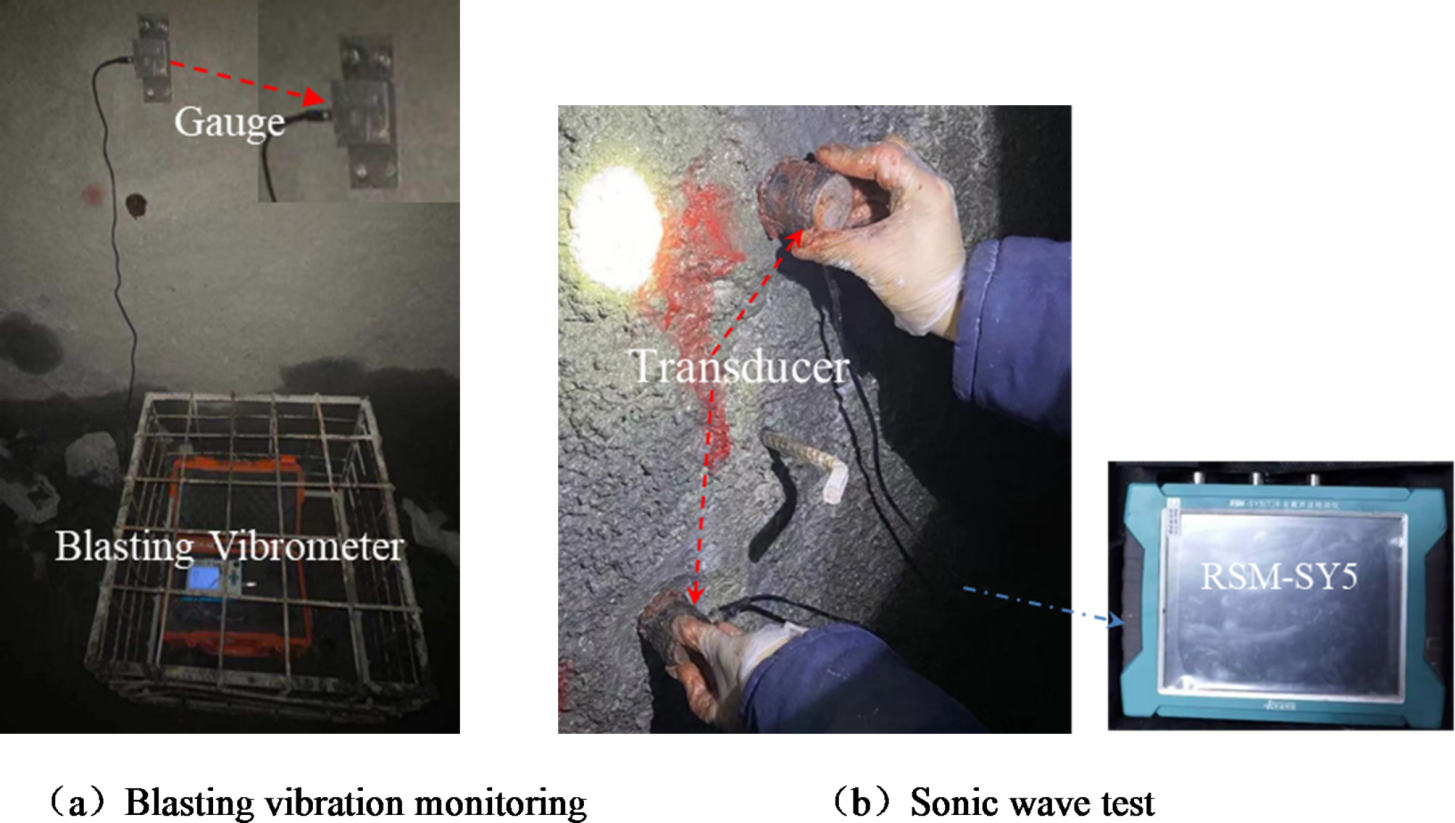
The specific layout of the site measuring points.

To reduce the test error, acoustic wave tests were carried out at 0.1 m on both sides of the vibration sensor, and the straight-line distance between the two horizontal transducers was 0.2 m. In the experiment, butter was used as a transducer coupling agent to keep the transducer close to the wall surface to improve the test effect. After each blasting construction, three parallel ultrasonic tests are carried out, and the average value of the three test results is used as monitoring result. The specific on-site arrangement of blasting vibration monitoring and acoustic wave testing is shown in Fig. 4.

Based on the above description, the monitoring points corresponding to the blasting vibration monitoring and the acoustic wave test are located at the same position. In other words, the *R* corresponding to the blasting vibration measurement point and the acoustic wave measurement point is approximately equal. As the tunneling progresses, the distances from the explosion corresponding to the vibration measuring points and the sound wave measuring points also change. During on-site monitoring, the laser rangefinder can be used to measure the distance from the explosion corresponding to each blasting operation, which facilitates subsequent analysis and calculation.

## Field test data analysis

### Analysis of blasting vibration monitoring data

**Fig. 5:**
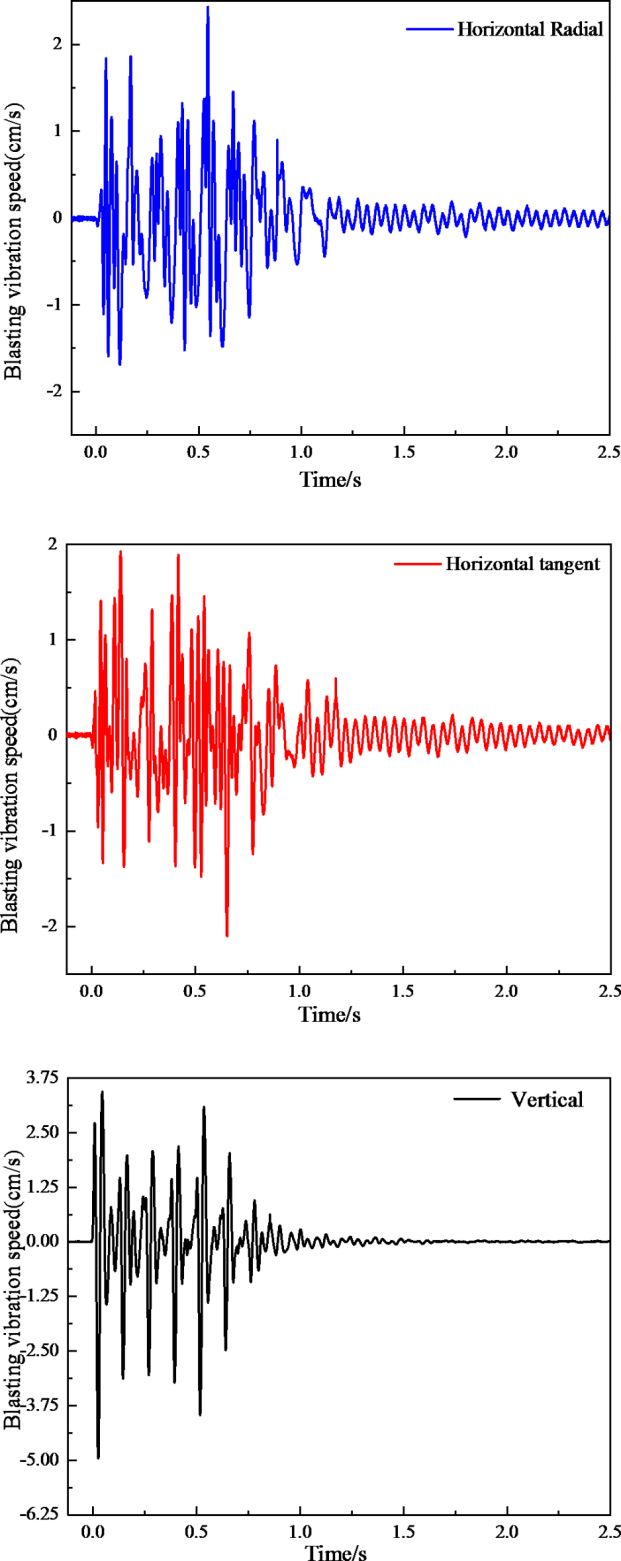
Typical blasting vibration time-history curve.

As shown in Fig. 5, compared with the *PPV* in the three directions in the blasting vibration time history curve, the blasting vibration velocity corresponding to the vertical direction is the largest. According to the requirements of relevant specifications^34–38^, this paper conducts subsequent research on the *PPV* corresponding to the vertical direction.

A total of 6 blasting vibration field monitoring tests were conducted from October 12 to October 21, 2018. Since the surrounding rock grade corresponding to the blasting construction of this section of the tunnel remained basically unchanged, the maximum charge per delay used in the blasting construction was 39.3 ~ 43.2 kg. At present, the Sadovsky formula is often used to reflect the attenuation law of blasting vibration, and the specific expression can be expressed as:

1 $$PPV = K\left( {\frac{{Q^{{\frac{1}{3}}} }}{R}} \right)^{\alpha }$$

where *PPV* represents the peak particle velocity, *Q* represents maximum charge per delay, *R* represents the distance between the measuring point and the blasting source, and *K* and *α* represent the geological parameters and attenuation parameters related to the blasting vibration transmission, respectively.

**Fig. 6:**
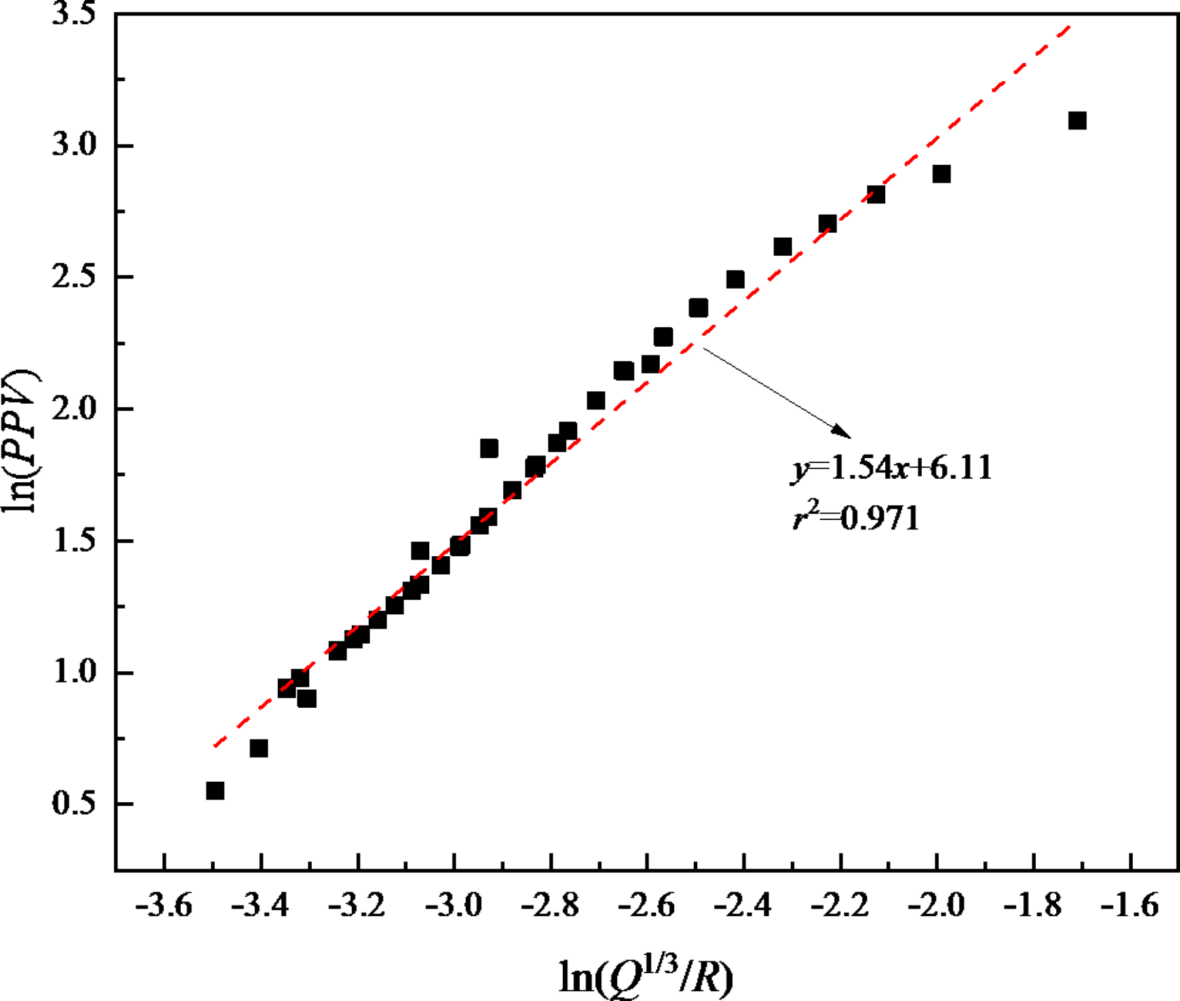
Summary and fitting curve of blasting vibration velocity.

Based on the field measured data, according to formula (1), regression fitting is performed and the fitting results are shown in Fig. 6. The specific fitting equation is expressed as follows:

2 $$PPV = 450.34\left( {\frac{{Q^{{\frac{1}{3}}} }}{R}} \right)^{{1.54}}$$

As shown in Fig. 6, the determination coefficient (*r*^2^) corresponding to formula (2) is 0.980, with high fitting accuracy, which can accurately reflect the blasting vibration attenuation law of the initial support concrete of the tunnel.

### Analysis of blasting sound wave test results

Considering the composition of concrete, primary cracks will exist on the surface of cement mortar, the interface between mortar and aggregate, and the surface of aggregate. Under the action of blasting vibration, the initial support concrete will first produce stress concentration at the tips of these cracks and cause local damage. As the number of blasting times increases, the number of microcracks gradually increases, and finally expands into macrocracks, resulting in a significant reduction in the bearing capacity of the concrete. At this point, if further damage effects occur, the concrete will suddenly break and fail.

In the process of blasting damage research, the cumulative damage variable *D*_*i*_ is often used to represent the degree of damage.

3 $${D_i}=1 - \frac{{{E_{mi}}}}{{{E_{m0}}}}$$

where *E*_*m*0_ represents the elastic modulus of the rock mass before the initial support concrete blasting, and *E*_*mi*_ represents the elastic modulus of the rock mass after the *i*-th blasting.

The Technical Specification for Excavation of Rock Foundations of Hydraulic Structures (SI47-2020) (2020) stipulates that the corresponding longitudinal wave velocity change rate before and after blasting should be used as the basis for evaluation.

4 $${\eta _i}=1 - \frac{{{C_{pi}}}}{{{C_{p0}}}}$$

where *η*_*i*_ represents the rate of change of the longitudinal wave velocity of the rock mass before and after blasting construction, *C*_*pi*_ represents the longitudinal wave velocity of the rock mass corresponding to the *i*-th blasting, and *C*_*p*0_ represents the longitudinal wave velocity of the rock mass corresponding to the blasting.

According to the elastic mechanics hypothesis, there is a certain functional relationship between the elastic modulus of rock mass and its longitudinal wave velocity. There is the following mathematical relationship between the blasting damage variable *D*_*i*_ and the rate of change of the longitudinal wave velocity *η*_*i*_.

5 $${D_i}=1 - {(1 - \eta )^2}$$

where *D*_*i*_ represents the cumulative damage variable corresponding to the *i*-th blasting.

According to the “Technical Specifications for Construction of Rock Foundation Excavation Engineering of Hydraulic Structures” (SI47-2020), when *η* < 0.1, the rock mass is considered to be undamaged or slightly damaged; when 0.1 < *η* ≤ 0.15, the rock mass is considered to be slightly damaged; when *η* > 0.15, the rock mass is considered to be damaged or the excavated rock mass is of poor quality. That is, *η* = 0.1 can be used as the control threshold, and the corresponding critical value of rock mass cumulative damage *D*_*cr*_ = 0.19.

Before blasting construction, three acoustic wave tests were carried out near each vibration measuring point to obtain the corresponding longitudinal wave velocity, and the average value of the three test results was taken as *C*_*p*0_. Within 24 h after each blasting construction, the longitudinal wave velocity of the corresponding initial support concrete is measured at the same position, and the corresponding longitudinal wave velocity *C*_*pi*_ after each blasting can be obtained, *i* = 1,2…,6. The results of the acoustic wave test are shown in Table 4.

**Table 4:** Acoustic monitoring data of each measuring point.

Measuring point	Longitudinal wave speed /(m۰s^-1^)						
	C_p0_	C_p1_	C_p2_	C_p3_	C_p4_	C_p5_	C_p6_
1	1400	1261	1235	1200	1183	1155	1145
2	1450	1323	1302	1273	1258	1235	1215
3	1361	1262	1248	1225	1214	1196	1178
4	1388	1310	1300	1281	1273	1259	1243
5	1305	1255	1248	1237	1230	1223	1211
6	1279	1254	1251	1245	1242	1238	1233

Based on the data in Table 4, the cumulative damage *D*_*i*_ is obtained through formulas (3 ~ 5) as shown in Table 5. It can be seen from Table 5 that as the number of blasting increases, the blasting cumulative damage variables corresponding to each measuring point gradually increase. In addition, the closer the measuring point is to the blasting source, the greater the cumulative damage. After six blasting experiments, the cumulative damage corresponding to measuring point 1 is as high as 0.331, which has exceeded the critical damage threshold of 0.19. In addition, the cumulative damage (*D*) corresponding to measuring points 2, 3, and 4 also exceeds the control threshold. However, it can be found that with the increase in the number of blasting times, the damage increment tends to gradually decrease, which shows that the damage effect induced by blasting vibration does not cause sudden fracture and damage of the initial supporting concrete.

**Table 5:** Damage increment and cumulative damage before and after blasting.

Measuring point	Cumulative damage D					
	I	II	III	IV	V	VI
1	0.189	0.221	0.265	0.286	0.319	0.331
2	0.168	0.193	0.229	0.247	0.275	0.298
3	0.140	0.159	0.190	0.205	0.228	0.251
4	0.109	0.123	0.148	0.159	0.177	0.198
5	0.075	0.085	0.102	0.111	0.122	0.140
6	0.039	0.044	0.053	0.057	0.063	0.071

**Fig. 7:**
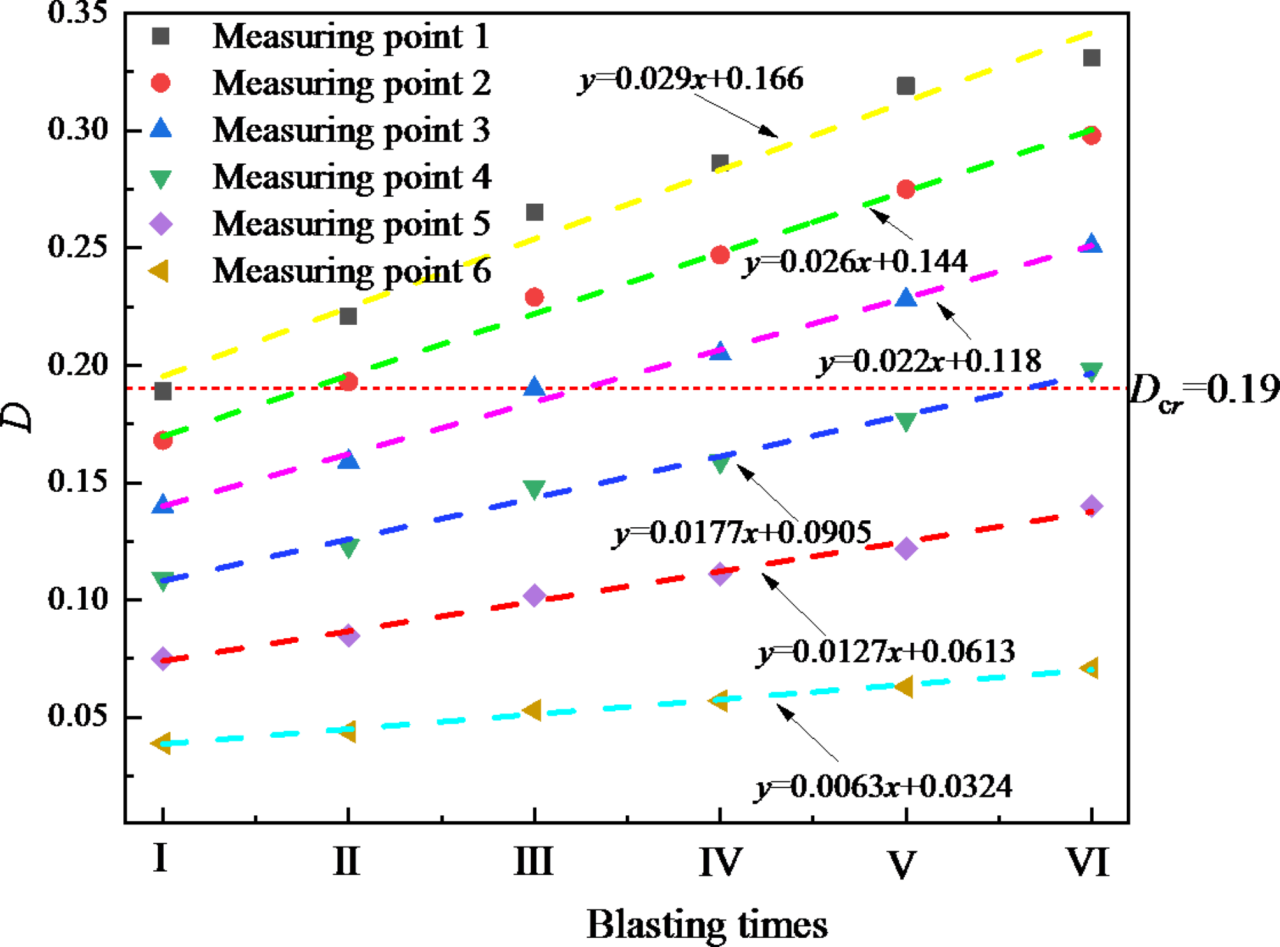
Relationship between blasting damage variables and blasting times.

In order to more clearly analyze the changes in blasting damage variables, the relationship between the cumulative damage variable *D*_*i*_ corresponding to each blasting construction and the number of blasting times is plotted in Fig. 7. As shown in Fig. 7, the cumulative damage variable and the number of blasting times are approximately monotonous linear. As the blasting distance increases, the corresponding fitting straight line slope decreases from 0.029 to 0.0063, and the change rate of the damage variable shows a gradually decreasing trend. Taking *D*_*cr*_=0.19 as the criterion, it can be found that as the number of blasting increases, the cumulative damage variables corresponding to multiple measuring points exceed 0.19. Among them, measuring point 1 is the shortest distance from the blasting source, and the cumulative damage variable corresponding to the second blasting has exceeded 0.19, which is significantly affected by the blasting vibration damage.

## Cumulative damage characteristics of initial support concrete

**Fig. 8:**
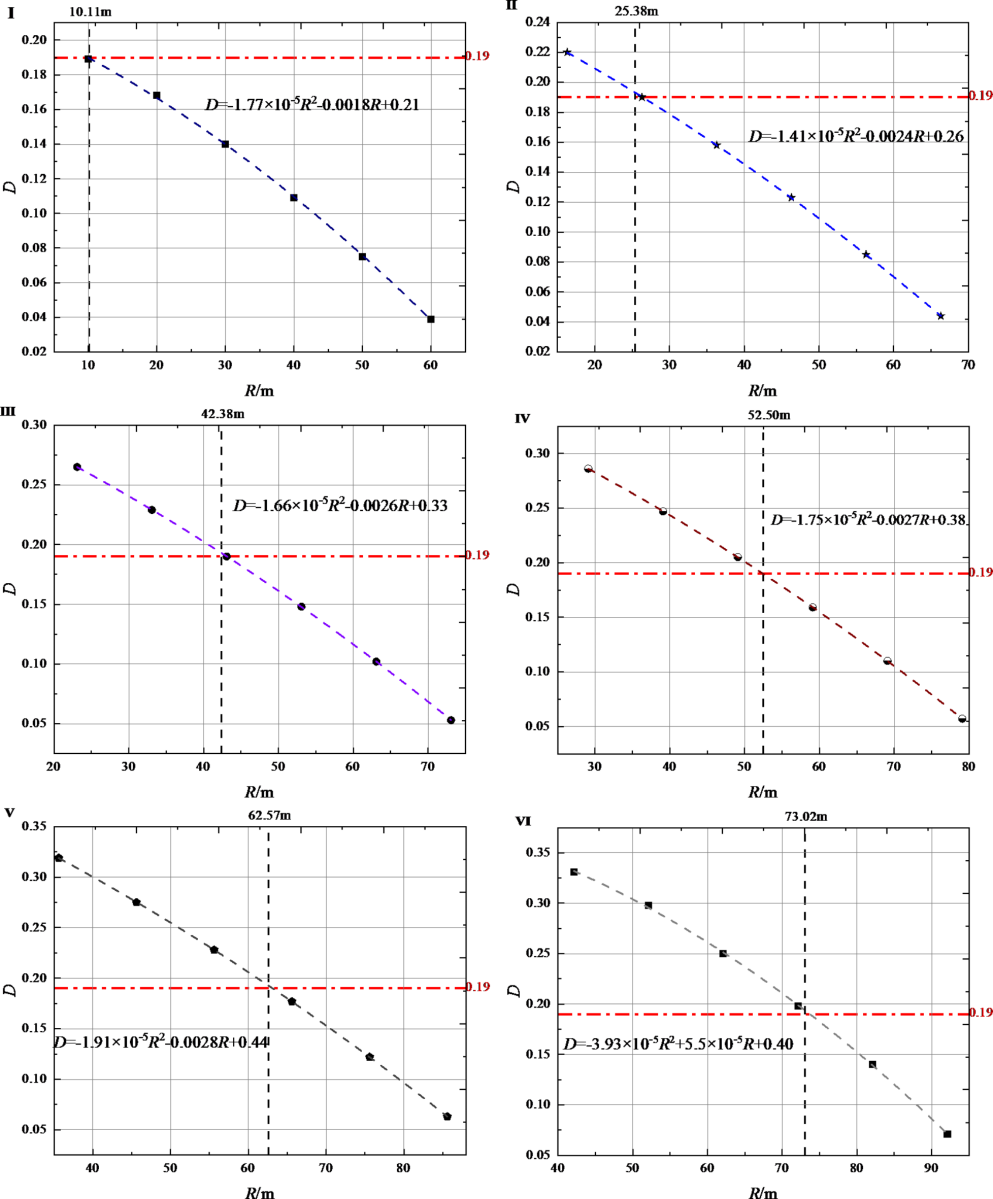
Relationship between cumulative damage variable *D* and blasting distance *R* corresponding to blasting experiments.

The analysis results in Sect. "Field test data analysis" show that the damage effect of the initial support of the tunnel is significant under the blasting load. However, the impact scope of blasting damage and specific control standards are not clearly defined. Therefore, this section systematically studies the cumulative damage characteristics of initial support through theoretical analysis methods, in order to obtain the threshold standard suitable for tunnel blasting damage control. First, the *R*-*D* scatter plots corresponding to each blasting test are drawn based on the data in Table 5. Among them, I~VI represent the first to sixth blasting tests respectively. As shown in Fig. 8, the cumulative damage variable *D* corresponding to the six blasting tests and the blasting distance *R* all show a good polynomial function relationship. We obtained six blasting tests using *D*_*cr*_ = 0.19 as the criterion, and the damage ranges *R*_*cr*_ corresponding to the initial support were 10.11 m, 25.38 m, 42.38 m, 52.50 m, 62.57 m and 73.02 m respectively. It can be seen that with the increase in the number of blasting times, the blasting damage range corresponding to the initial support of the tunnel gradually increases. This phenomenon is caused by the influence of the cumulative damage caused by blasting.

Since the charge used in the blasting construction of this section of the tunnel remains basically unchanged, the *PPV* can be expressed as a function of the blasting distance. Through the curve fitting method, the relationship between *PPV* and *R* is obtained as follows:

6 $$PPV=189.68{R^{ - 0.85}}$$

**Fig. 9:**
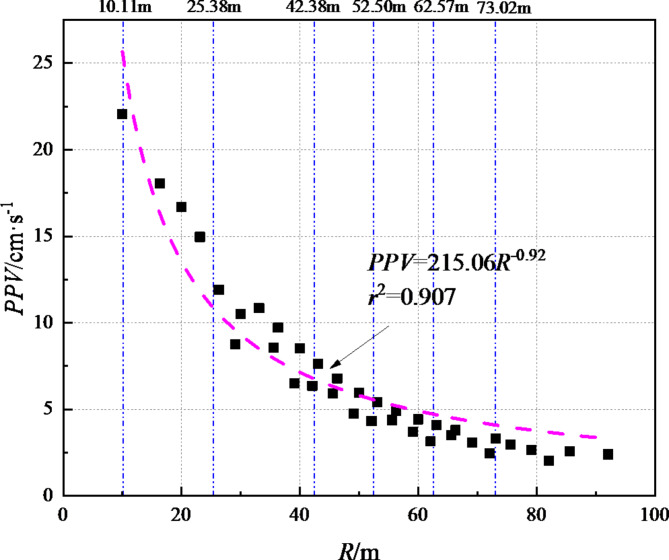
Scatter plot and mathematical relationship between *PPV* and blasting distance *R*.

Figure 9 shows that the determination coefficient (*r*^2^) corresponding to formula (6) is 0.907, and the fitting accuracy is relatively high. Through the *R*_*cr*_ obtained by the above calculation, we can get the *PPV*_*cr*_ corresponding to the critical damage position is 26.55 cm/s, 12.14 cm/s, 7.85 cm/s, 6.54 cm/s, 5.63 cm/s and 4.94 cm/s respectively.

A large number of research results^39–41^ show that the critical blasting damage distance is an important engineering parameter in blasting engineering and directly affects the calculation of blasting damage range. Currently, the critical blasting damage distance of tunnel rock mass or initial support is mostly obtained by using acoustic wave testing or numerical simulation. However, acoustic wave testing is significantly affected by the on-site testing environment, and the monitoring work is difficult, so the monitoring effect is not easy to guarantee. The accuracy of numerical simulation results depends on the on-site test results and the selection of geotechnical engineering parameters. The calculation is time-consuming and not easy to be used by engineering practitioners. Relevant engineering practice and research^42–44^ show that under the influence of blasting power, the critical damage distance (*R*_*cr*_) and the critical damage particle peak velocity (*PPV*_*cr*_) show a good nonlinear relationship. Compared with the sonic wave test, the blasting vibration test is simple and easy to perform.

**Fig. 10:**
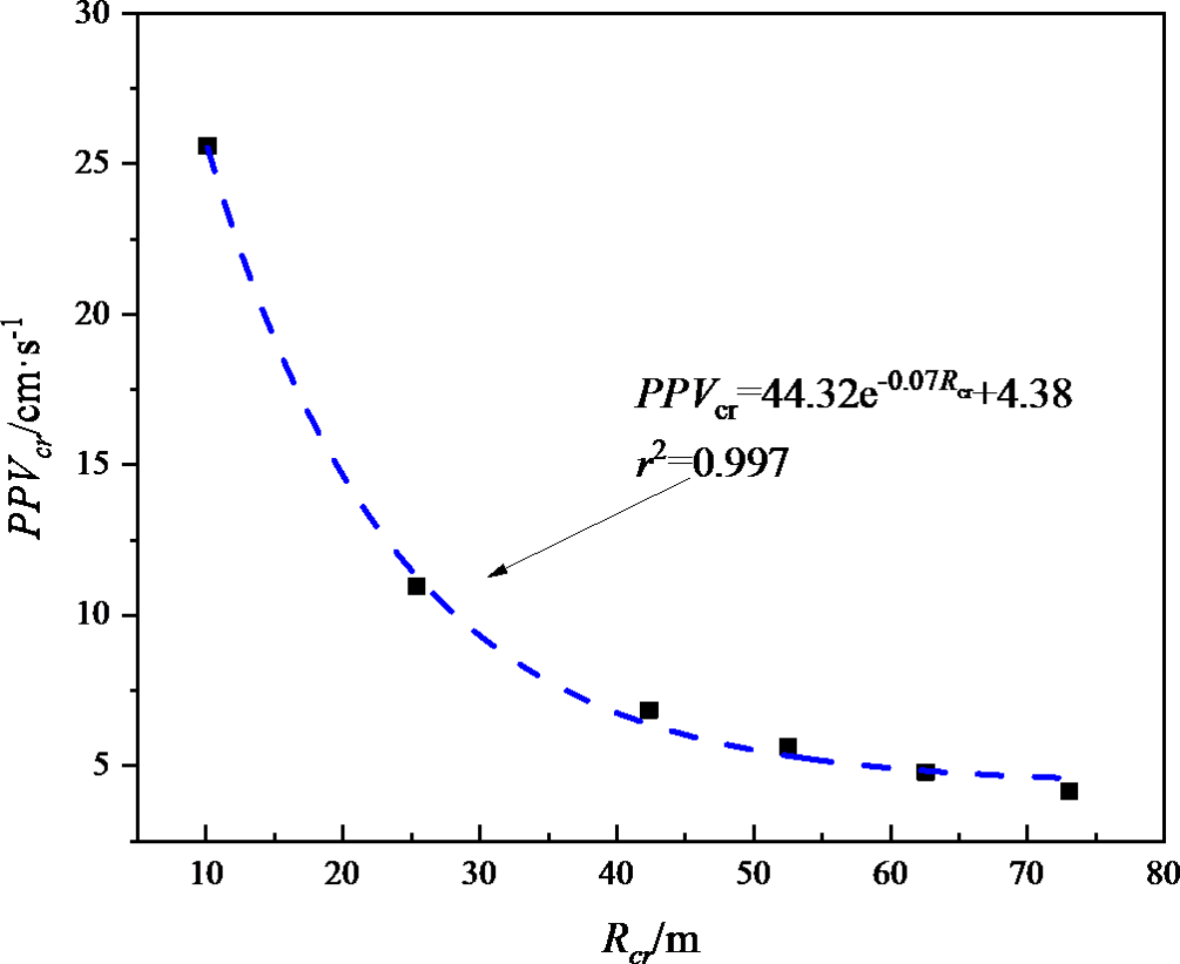
Scatter diagram and mathematical relationship between critical damage distance *R*_*cr*_ and corresponding vibration velocity *PPV*_*cr*_.

Therefore, it is worthwhile to explore in depth whether the damage range of the initial support of the tunnel can be controlled by controlling the value of the critical damage particle peak velocity (*PPV*_*cr*_). Therefore, the correlation data of critical damage distance (*R*_*cr*_) and critical damage particle peak velocity (*PPV*_*cr*_) are plotted as a scatter plot as shown in Fig. 10. The data fitting results in Fig. 10 show that there is a good exponential function relationship between *PPV*_*cr*_ and *R*_*cr*_, with a determination coefficient as high as 0.997. From the above analysis, it can be seen that, for this tunnel project, the purpose of quantitatively controlling the scope of blasting damage of the initial support of the tunnel can be achieved by setting the corresponding blasting vibration velocity threshold range.

For example, if the actual project requires that the blasting cumulative damage range of the initial support of the tunnel should not exceed 50 m, then it can be calculated that *R*_*cr*_ = 50 m corresponds to *PPV*_*cr*_ = 5.72 cm/s. That is, when the *PPV* is less than or equal to 5.72 cm/s, the initial support damage range of the tunnel caused by cyclic blasting is less than or equal to 50 m.

## Conclusion

Relying on the Caomaoshan Tunnel Project of Beijing-Zhangjiakou High-Speed Railway, blasting vibration monitoring and acoustic wave testing were carried out. This paper obtained the blasting vibration attenuation law of the initial support of the tunnel and the distribution characteristics of the blasting cumulative damage. Through data fitting, the article organically combines blasting vibration and cumulative damage, hoping to achieve the purpose of quantitatively controlling the scope of blasting damage in the initial support of the tunnel by setting the corresponding blasting vibration velocity threshold.

1. As the number of blasting increases, the cumulative damage of the initial support of the tunnel increases. As the blasting distance increases, the increment of the cumulative damage shows a trend of gradually decreasing, and the corresponding fitting line slope of the blasting cumulative damage decreases from 0.029 to 0.0063.
2. Taking *D*_*cr*_=0.19 as the control standard, the blasting cumulative damage ranges (*R*_*cr*_) corresponding to each blasting test were 10.11 m, 25.38 m, 42.38 m, 52.50 m, 62.57 m and 73.02 m respectively. Combined with the attenuation law of blasting vibration, the critical vibration velocities (*PPV*_*cr*)_ of blasting damage are obtained to be 26.55 cm/s, 12.14 cm/s, 7.85 cm/s, 6.54 cm/s, 5.63 cm/s and 4.94 cm/s respectively.
3. The numerical analysis results show that there is a good exponential function relationship between the blasting damage range (*R*_*cr*_) and the corresponding blasting damage critical vibration velocity (*PPV*_*cr*_). In the early stage when the surrounding rock grade, rock mass lithology and blasting footage remain basically unchanged, the purpose of quantitatively controlling the cumulative damage range of the initial support blasting of the tunnel can be achieved by setting the corresponding blasting vibration velocity control threshold.

## Data Availability

If necessary, the raw data in the manuscript can be obtained by contacting the first author or corresponding author.

## Abbreviations

*PPV*: Peak particle velocity
*σ*_*c*_: Uniaxial compressive strength
*R*: Distance from the blast area
*Q*: Maximum charge per delay
*R*_*cr*_: Distance corresponding to critical damage
*PPV*_*cr*_: Peak particle velocity corresponding to critical damage
*D*: Cumulative damage
